# Macon Medical Society

**Published:** 1889-04

**Authors:** J. A. Toole

**Affiliations:** Reporter


### MACON MEDICAL SOCIETY.

February, 1889.

The President, Dr. N. G. Gewiner, in the chair, Dr. H. McHatton Secretary. The following gentlemen were present: Drs. K. P. Moore, W. R. Winchester, W. S. Holt, J. A. Toole, H. J. Williams, J. C. Johnston. W. C. Gibson, R. O. Cotter, J. T. Doory.

The subject for discussion, "The primary dressing of wounds," being announced by the President, a very interesting ventilation of the subject ensued, nearly all present taking a part, giving their views and personal experiences. Incised, contused, lacerated and gun-shot wounds, and the usual mode adopted by each in the dressing of same.. Coaptation, union by first intention, and antiseptics were discussed. The applications most generally employed being carbolic acid, bichloride mercury, iodoform and colodion, though oil turpentine as a dressing was not without its advocates.

The President now called for reports of new cases, when Dr. H. J. Williams said he had recently under treatment a case of sciatica, in which the usual remedies failed, and as he had occasion to suspect some venereal trouble, he made an examination, and found a contracted meatus. He opened the meatus and dilated the urethra, after which the sciatica disappeared. Dr. K. P. Moore stated that he had seen similar cases, and no doubt many urethral discharges had their origin and continuance in this cause. Dr. H. McHatton said he had lately operated on a case of phymosis that resulted in the formation of a keloid. Dr. K. P. Moore said he had a case of ovariatomy to report. The tumor was a large one, with many adhesions, but the case had done well from first to last. The wound healed nicely, and the temperature was never very great.

The Society having gotten up a black list for the purpose of knowing who was in the habit of paying medical bills, those present having their list prepared, handed in the same to the Secretary and the meeting adjourned.

J. A. TOOLE, Reporter.

